# Supplementary material for: Adjusted Comparison of Outcomes between Patients from CARTITUDE-1 versus Multiple Myeloma Patients with Prior Exposure to PI, Imid and Anti-CD-38 from a German Registry
Source: Cancers (Basel). 2021 Nov 29;13(23):5996. doi: 10.3390/cancers13235996 (PMC8656798; doi:10.3390/cancers13235996)
Supplement: Supplementary file 1 [file cancers-13-05996-s001.zip › cancers-1408767-supplementary.pdf]

cancers MDPI

Adjusted comparison of outcomes between patients from CARTITUDE-1 versus multiple myeloma patients with prior exposure to PI, IMiD and anti-CD-38 from a German registry”

Maximilan Merz, Hartmut Goldschmidt, Parameswaran Hari, Mounzer Agha, Joris Diels, Francesca Ghilotti, Nolen J Perualila, Jedelyn Cabrieto, Benjamin Haefliger, Henrik Sliwka, Jordan M Schecter, Carolyn C Jackson, Yunsi Olyslager, Muhammad Akram, Tonia Nesheiwat, Lenka Kellermann, Sundar Jagannath

Text S1: Additional Details, Study Methods

Deriving R-ISS for CARTITUDE-1

R-ISS was not collected as a variable in CARTITUDE-1. It was derived based on available variables as follows:

- If ISS=1 and LDH low and no cytogenetic abnormalities/not tested, then R-ISS=1
- If ISS=3 and (LDH high or cytogenetic abnormalities), then R-ISS=3
- Else R-ISS=2

Note In CARTITUDE-1, LDH values were complete.

Deriving R-ISS in TherapieMonitor Data Source

R-ISS was not directly available as a covariate within the TherapieMonitor eCRF maintained by the OncologyInformationService. For the TherapieMonitor Cohort R-ISS was defined as follows:

- If ISS=1 and LDH low and no cytogenetic abnormalities/not tested, then R-ISS=1
- If ISS=1 and LDH missing and no cytogenetic abnormalities/not tested, then R-ISS=1
- If ISS=3 and (LDH high or cytogenetic abnormalities), then R-ISS=3
- If ISS=3 and (LDH missing and no cytogenetic abnormalities/not tested), then R-ISS=3
- Else R-ISS=2

Handling of Multiple Lines Per Patient

Hernán et al.[25] suggest using a single eligible time (e.g., the first, the last, or a random eligible time), or all eligible times or a subset thereof following unbiased options for choosing T0. The latter strategy requires emulating multiple nested trials, each with a different start of follow-up, which can be more statistically efficient. Because individuals may be included in multiple emulated trials, appropriate adjustment of the usual variance estimator is required. Using the former strategy and choosing either the first eligible index date or the last eligible index date as T_0 may lead to bias since death close to the first eligible time may be due to factors other than the care received, and death is more likely among patients receiving last LOT.

Overview: Study Populations, Eligibility and Dates

Population	Description
CARITUDE-1 ITT	All enrolled patients; Index date: date of apheresis
CARITUDE-1 mITT	All infused patients; Index date: date of infusion
RWCP ITT	All LOTs; Index date: date of treatment initiation of LOT
RWCP mITT	All LOTs, excluding LOTs from patients with an event or follow-up censored within 52 days since treatment initiation (mean time from apheresis to infusion); Index date: date of treatment initiation plus 52 days

Table S1 : Demographics Pre- and Post-Weighting

Overview of Group Demographic Balance Before and After IPW-ATT and IPW-ATO Weighting (mITT Population; All Index Dates)

Variable	Categories	Before IPW (mITT)			After IPW-ATT Weighting (mITT)			After IPW-ATO Weighting (mITT)		
		CARTITUDE -1, % (N=97)	TherapieMonitor Cohort, % (N=223)	SMD	CARTITUDE -1, % (N=97)	TherapieMonitor Cohort, % (N=42)	SMD	CARTITUDE -1, % (N=13)	TherapieMonitor Cohort, % (N=13)	SMD
**Refractory status**	Penta refractory<br>Quad refractory<br>Triple refractory<br><= Double refractory	42.3%<br>37.1%<br>3.2%<br>12.4%	0.4%<br>6.7%<br>11.7%<br>81.2%	2.34	42.3%<br>37.1%<br>8.2%<br>12.4%	20.6%<br>31.9%<br>9.5%<br>37.9%	0.71	6.7%<br>26.7%<br>20.6%<br>46.0%	6.7%<br>26.7%<br>20.6%<br>46.0%	0
**R-ISS stage**	I<br>II<br>III	34.0%<br>58.8%<br>7.2%	1.3%<br>42.6%<br>56.1%	1.50	34.0%<br>58.8%<br>7.2%	20.7%<br>54.0%<br>25.3%	0.54	15.4%<br>65.8%<br>18.7%	15.4%<br>65.8%<br>18.7%	0
**Time to progress on last trt (months)**	< 4<br>4+	49.5%<br>50.5%	21.1%<br>78.9%	-0.62	49.5%<br>50.5%	54.2%<br>45.8%	0.10	35.8%<br>64.2%	35.8%<br>64.2%	0
**Number of prior LOTs**	≤ 4<br>5+	34.0%<br>66.0%	86.5%<br>13.5%	1.27	34.0%<br>66.0%	35.7%<br>64.3%	0.03	54.3%<br>45.7%	54.3%<br>45.7%	0
**ECOG status**	0<br>1	40.2%<br>59.8%	11.2%<br>88.8%	-0.70	40.2%<br>59.8%	10.3%<br>89.7%	-0.73	16.6%<br>83.4%	16.6%<br>83.4%	0
**Age (years)**	<65<br>65 to <75<br>75+	63.9%<br>24.7%<br>11.3%	14.3%<br>49.8%<br>35.9%	1.21	63.9%<br>24.7%<br>11.3%	51.5%<br>38.0%<br>10.5%	0.28	41.9%<br>39.5%<br>18.5%	41.9%<br>39.5%<br>18.5%	0
**Sex**	Male<br>Female	58.8%<br>41.2%	61.9%<br>38.1%	0.06	58.8%<br>41.2%	47.9%<br>52.1%	-0.22	56.3%<br>43.7%	56.3%<br>43.7%	0
**Years since diagnosis**	<6<br>6 +	46.4%<br>53.6%	85.7%<br>14.3%	0.91	46.4%<br>53.6%	41.8%<br>58.2%	-0.09	46.3%<br>53.7%	46.3%<br>53.7%	0
**Average duration of prior lines (months)**	<8.14<br>8.14 to <11.76<br>11.76 to <17.61<br>>17.61 months	20.6%<br>22.7%<br>27.8%<br>28.9%	15.2%<br>33.2%<br>34.5%<br>17%	0.36	20.6%<br>22.7%<br>27.8%<br>28.9%	22.6%<br>7.9%<br>24.2%<br>45.3%	0.50	12.1%<br>15.2%<br>30.7%<br>42.0%	12.1%<br>15.2%<br>30.7%<br>42.0%	0
**Summary Diagnostics**										
	**Mean SMD**	1.00			0.36			0		
	**% SMDs > 0.2**	8/9 = 88.9%			6/9 = 66.6%			0/9 = 0%		

Note R-ISS is derived for both CARTITUDE-1 and TherapieMonitor

Abbreviations: SMD, standardized mean difference; IPW, inverse probability weighting; ATT: average treatement effect on the treated; ATO: overlap weighting

Table S2: Treatment Regimens, External Control Group

A total of 33 unique treatment regimens were received by patients from the German external control group represented by the TherapieMonitor cohort. Details of the frequency of each regimen are provided below.

Treatment Regimen	Number of Lines of Treatment
IxaRd (ixazomib, lenalidomide, dexamethasone)	55 (17.6%)
Pd (pomalidomide, dexamethasone)	47 (15.1%)
MP (melphalan, prednisone)	33 (10.6%)
EloRd (elotuzumab, lenalidomide, dexamethasone)	23 (7.4%)
Daratumumab	22 (7.1%)
Vd (bortezomib, dexamethasone)	22 (7.1%)
TP (thalidomide, prednisone)	13 (4.2%)
Kd (carfilzomib, dexamethasone)	13 (4.2%)
PanoVd (panobinostat, bortezomib, dexamethasone)	13 (4.2%)
DVd (daratumumab, bortezomib, dexamethasone)	11 (3.5%)
Benda-Pred (bendamustine, prednisone)	8 (2.6%)
EloPd (elotuzumab, pomalidomide, dexamethasone)	8 (2.6%)
Rd (lenalidomide, dexamethasone)	7 (2.2%)
KRd (carfilzomib, lenalidomide, dexamethasone)	6 (1.9%)
DRd (daratumumab, lenalidomide, dexamethasone)	5 (1.6%)
PVd (pomalidomide, bortezomib, dexamethasone)	4 (1.3%)
PCd (pomalidomide, cyclophosphamide, dexamethasone)	3 (1%)
Bendamustin	2 (0.6%)
Elo mono (elotuzumab)	2 (0.6%)
KCd (carfilzomib, cyclophosphamide, dexamethasone)	2 (0.6%)
Other-Dex (other treatment, dexamethasone)	1 (0.3%)
Doxorubicin	1 (0.3%)
Pano-Dex (panobinostat, dexamethasone)	1 (0.3%)
DPd (daratumumab, pomalidomide, dexamethasone)	1 (0.3%)
R (lenalidomide)	1 (0.3%)
Elo-Ixa-Len (elotuzumab, ixazomib, lenalidomide)	1 (0.3%)
DKd (daratumumab, carfilzomib, dexamethasone)	1 (0.3%)
KRP (carfilzomib, lenalidomide, pomalidomide)	1 (0.3%)
Velcade (bortezomib)	1 (0.3%)
VC (bortezomib, cyclophosphamide)	1 (0.3%)
DPVd + adriamycin (daratumumab, pomalidomide, bortezomib, dexamethasone, adriamycin)	1 (0.3%)
VRCd (bortezomib, lenalidomide, cyclophosphamide, dexamethasone)	1 (0.3%)
VTd (bortezomib, thalidomide, dexamethasone)	1 (0.3%)

Text S2: Definitions of Refractory Disease

With regard to disease status, the following definitions were employed for types of refractory disease in the analyses presented:

- **Tri-refractory**: Refractory to an immunomodulatory drug (IMiD; either lenalidomide, pomalidomide, thalidomide), a proteasome inhibitor (PI; either bortezomib, carfilzomib, ixazomib) and an anti-CD38 MoAb (either daratumumab, isatuximab)
- **Quadruple refractory**: Refractory to either two IMiDs, one PI and at least one anti-CD38 mAb OR one IMiD, two PIs and at least one anti-CD38 MoAb
- **Penta-refractory**: Refractory to two IMiDs, two PIs and at least one anti-CD38 MoAb.

Figure S1: Covariate Effects from Cox Regression, TTNT, mITT cohorts

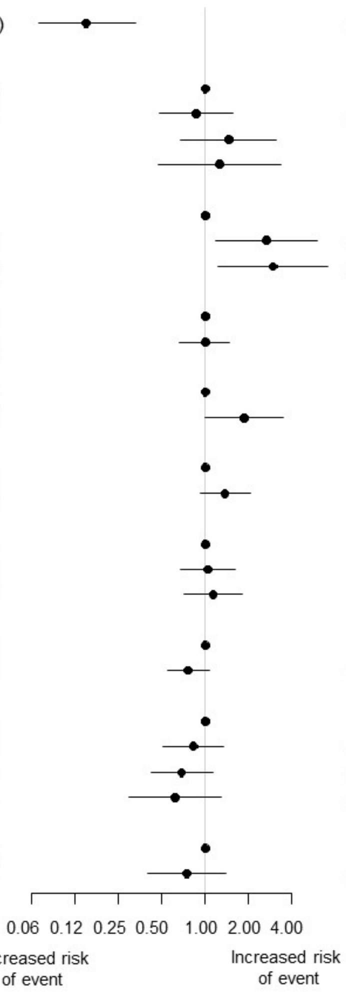

Variable	Variable Category	N CARTITUDE (%)	N RWCP (%)	HR	(95% CI)
Trt effect (CAR-T vs RWCP)		97 (100%)	223 (100%)	0.15	(0.07, 0.33)
Refractory Status					
	<=Double refractory (ref)	12 (12%)	181 (81%)	1.00	(1.00, 1.00)
	Triple refractory	8 (8%)	26 (12%)	0.86	(0.48, 1.54)
	Quadruple refractory	36 (37%)	15 (7%)	1.45	(0.67, 3.13)
	Penta-refractory	41 (42%)	1 (0%)	1.25	(0.47, 3.35)
R-ISS Stage					
	I (ref)	33 (34%)	3 (1%)	1.00	(1.00, 1.00)
	II	57 (59%)	95 (43%)	2.67	(1.19, 5.99)
	III	7 (7%)	125 (56%)	2.96	(1.22, 7.18)
TTP, prior LOT					
	<4 months (ref)	48 (49%)	47 (21%)	1.00	(1.00, 1.00)
	4+ months	49 (51%)	176 (79%)	1.00	(0.66, 1.49)
# prior LOTs					
	<=4 (ref)	33 (34%)	193 (87%)	1.00	(1.00, 1.00)
	5+	64 (66%)	30 (13%)	1.88	(1.00, 3.53)
ECOG Performance Status					
	0 (ref)	39 (40%)	25 (11%)	1.00	(1.00, 1.00)
	1	58 (60%)	198 (89%)	1.38	(0.92, 2.09)
Age (years)					
	<65 (ref)	62 (64%)	32 (14%)	1.00	(1.00, 1.00)
	65 to <75	24 (25%)	111 (50%)	1.04	(0.67, 1.60)
	75+	11 (11%)	80 (36%)	1.14	(0.71, 1.82)
Gender					
	Male (ref)	57 (59%)	138 (62%)	1.00	(1.00, 1.00)
	Female	40 (41%)	85 (38%)	0.76	(0.55, 1.06)
Average duration of prior LOTs					
	<8.14 months (ref)	20 (21%)	34 (15%)	1.00	(1.00, 1.00)
	8.14-<11.76 months	22 (23%)	74 (33%)	0.83	(0.51, 1.33)
	11.76-<17.61 months	27 (28%)	77 (35%)	0.69	(0.42, 1.13)
	17.61+ months	28 (29%)	38 (17%)	0.62	(0.30, 1.29)
Years since diagnosis					
	<6 years (ref)	45 (46%)	191 (86%)	1.00	(1.00, 1.00)
	6+ years	52 (54%)	32 (14%)	0.75	(0.40, 1.40)

Time to Next Treatment, mITT comparison, Covariate Effects from Cox PH Multivariable Regression Analysis. The clinical effects associated with the modeled covariates in a multivariable Cox proportional hazards regression model for TTNT are presented based upon data from the mITT population. R-ISS stage was derived for both cohorts based on individual components. Abbreviations: CI, confidence interval; LOT, line of therapy; HR, hazard ratio; R-ISS, Revised International Staging System; RWCP, real-world clinical practice.

Figure S2: Propensity Score Distributions

Propensity score distributions for (a) observed, (b) ATT weighted and (c) ATO weighted, ITT population

Prior to IPW Weighting (Naïve Comparison)

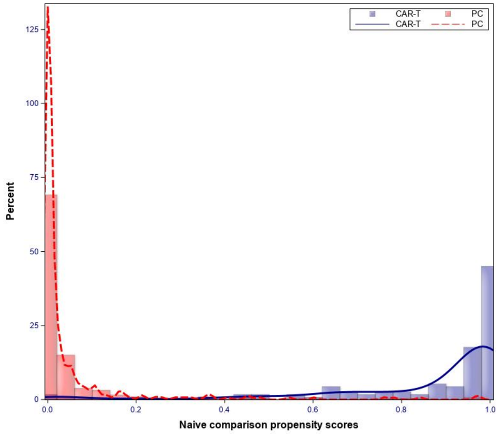

(a)

IPW-ATT Weighting

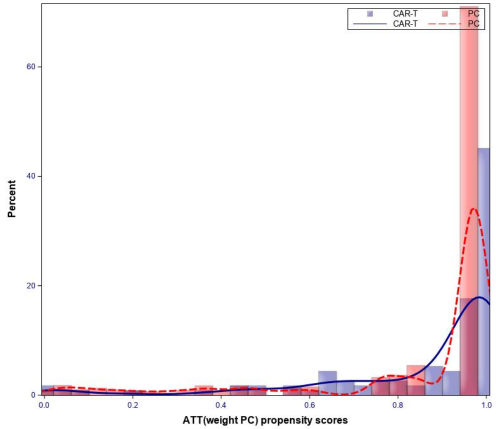

(b)

IPW-ATO Weighting

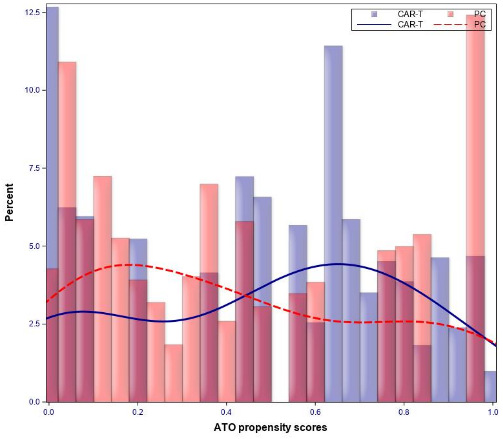

(c)

Note Y-axis scales differ between graphs

Propensity score distributions for (a) observed, (b) ATT weighted and (c) ATO weighted, mITT population

Prior to IPW Weighting (Naïve Comparison)

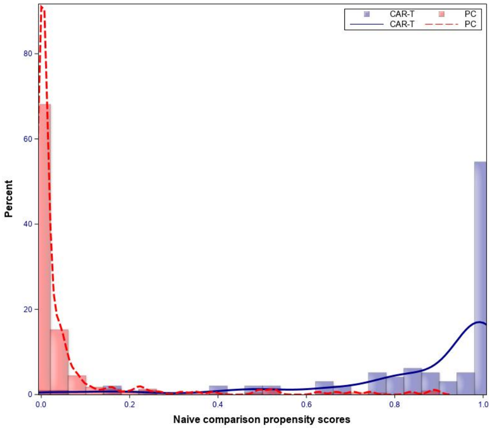

(a)

IPW-ATT Weighting

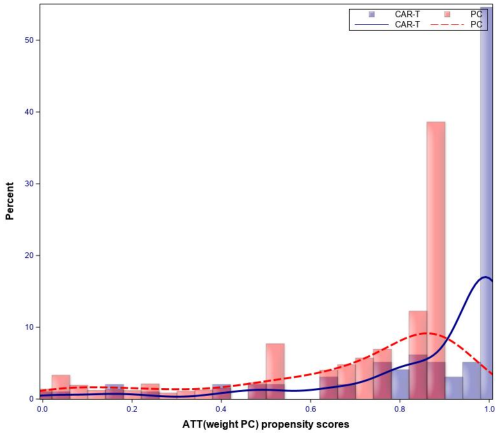

(b)

IPW-ATO Weighting

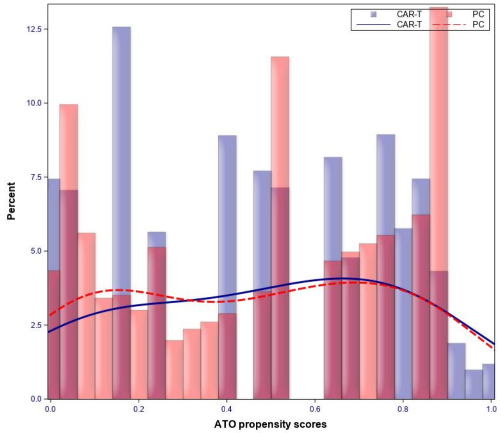

(c)

Note Y-axis scales differ between graphs
